# A Systematic Review of Microplastic Contamination in Tuna Species: General Pathways into the Food Chain with Ecotoxicological and Human Health Perspectives

**DOI:** 10.3390/foods14203547

**Published:** 2025-10-17

**Authors:** Leila Peivasteh-roudsari, Fardin Javanmardi, Parisa Shavali Gilani, Behrouz Tajdar-oranj, Zohreh Safayi Doost, Hananeh Yazdanbakhsh, Burhan Basaran

**Affiliations:** 1 Department of Environmental Health Engineering, Food Safety Division, School of Public Health, Tehran University of Medical Sciences, Tehran 11369, Iran; leila.peivasteh@yahoo.com (L.P.-r.); parisashavalii@gmail.com (P.S.G.); 2 Department of Food Science and Technology, School of Nutritional Sciences and Dietetics, Tehran University of Medical Sciences, Tehran 11369, Iran; f.javanmardy@gmail.com; 3 Research Center for Environmental Determinants of Health (RCEDH), Kermanshah University of Medical Sciences, Kermanshah 67146, Iran; 4 Department of Nutrition, School of Allied Medical Sciences, Ahvaz Jundishapur University of Medical Sciences, Ahvaz 61000, Iran; lsafsyi1988@gmail.com; 5 Student Research Committee, Kermanshah University of Medical Sciences, Kermanshah 67146, Iran; hana_yzb2001@yahoo.com; 6 Department of Nutrition and Dietetics, Faculty of Health Sciences, Recep Tayyip Erdogan University, Rize 53100, Türkiye

**Keywords:** food safety, tuna species, microplastic, risk evaluation, health

## Abstract

Tuna species, as highly migratory apex predators of major commercial significance, play a vital role as biological indicators of microplastics (MPs) contamination due to their trophic position and wide geographic distribution. Current systematic review aims to analyze the occurrence, characteristics, and concentrations of MPs in various tuna species. Data from 19 studies were compiled, focusing on the presence of MPs in different organs (gills, muscles, gastrointestinal tracts). High concentrations of MPs were found in tuna species from the Bay of Bengal (42.13 ± 13.58 MPs/individual in *Thunnus obesus*) and the Persian Gulf (5.71 MPs/individual in *Thunnus tonggol*), indicating significant contamination in these regions. Polyethylene (PE) and Polypropylene (PP) were the most commonly detected polymers, suggesting their widespread presence in marine environments. The dominant size range of MPs was 0.5–2.5 mm, with fibers and fragments being the most common shapes. The presence of MPs in edible tissues raises concerns about potential health risks for both marine life and human consumers. Future research should focus on expanding geographical coverage and investigating the ecological and health impacts of MPs ingestion. Long-term monitoring and international collaboration are essential to address this global environmental challenge effectively.

## 1. Introduction

Seafood is important for global nutrition, representing over 17% of animal protein intake worldwide [1,2]. Indigenous coastal communities have been found to consume seafood at a significantly higher rate, approximately 15 times greater, than non-Indigenous populations [3]. The widespread presence of plastic waste in marine environments poses a significant threat to seafood safety that warrants serious consideration [4]. Plastic waste constitutes 60–80% of marine debris, with PE and PP accounting for more than half of the global output, which has become a pervasive environmental crisis [5,6]. Since the 1950s, plastic production has experienced significant growth, culminating in a total of 390.7 million metric tons (Mt) in 2021, with projections estimating 1100 Mt by 2050 [7]. Despite widespread use of plastic in packaging (39.5%), construction (20.1%), and other industries, only 10% of plastic waste is recycled, while 76% is disposed of in landfills or the natural environment, ultimately entering aquatic ecosystems [8]. The persistence of plastics in the environment leads to their degradation into smaller particles, which are classified into three categories: mesoplastics (5 mm to 1 cm), microplastics (MPs; 0.1 µm to 5 mm), and nanoplastics (<0.1 µm) [9,10]. MPs originate from two primary sources: the primary MPs are specifically designed and manufactured for utilization in personal care products and various industrial applications, and secondary MPs are formed when larger plastic debris undergoes degradation through various processes, including exposure to ultraviolet (UV) radiation, biological factors, or mechanical actions [8,11,12]. These particles have become ubiquitous pollutants, detected in various forms (pellets, films, fibers, fragments, etc.) across aquatic organisms, from plankton to fish [13,14,15].

The ingestion of MPs by marine organisms poses significant ecological and health risks. Aquatic species encounter MPs through two primary pathways: direct ingestion of the particles and consumption of prey that has been contaminated [16]. Upon ingestion, MPs have the potential to accumulate in the gastrointestinal tract or to migrate to other organs within the body, causing physical damage, inflammation, and the release of toxic additives such as phthalates and bisphenol A [17,18]. This contamination raises concerns about bioaccumulation and biomagnification through the trophic chain, with documented impacts on 30–81 marine mammal species resulting from entanglement or ingestion. The persistence of MPs and their associated toxins also poses potential long-term risks to human health, particularly for populations reliant on seafood [19]. Despite these challenges, regulatory frameworks addressing MPs remain inadequate. The United States Food and Drug Administration (USFDA) lacks specific regulations for MPs [20], while the European Food Safety Authority (EFSA) has highlighted the need for standardized detection methods and toxicity evaluations [21]. The Food and Agriculture Organization (FAO) has also emphasized the absence of international laws targeting MPs in food. In response, the fifth session of the United Nations Environment Assembly (UNEA-5.2) endorsed a landmark resolution to develop an international, legally binding agreement by 2024 to address plastic pollution [7]. It is essential to address these research gaps, as the nutritional benefits of seafood consumption are crucial for health and well-being.

According to previous studies, pelagic fish, particularly tuna species, have had the highest concentration of MPs [22,23,24]. Tuna are exceptional bioindicators of marine pollution primarily because their unique biology and behavior lead to a greater accumulation of contaminants compared to most other fish. As active, high-trophic-level predators, they consume vast amounts of diverse prey from various depths, constantly ingesting pollutants that have biomagnified through the food chain [25]. Their extensive migratory patterns, both across oceans and through the entire water column (from surface to 1000 m deep), expose them to pollutants from a wide range of geographical areas and oceanic layers [26]. Furthermore, their high metabolic rate, endothermy, and long lifespans result in accelerated uptake and significant bioaccumulation of contaminants over time [27]. The combination of intense feeding behavior, extensive migratory patterns, and unique physiological traits positions tuna as exceptional biological indicators. Tuna species can provide a comprehensive assessment of marine ecosystem health and the distribution of contaminants within these environments [28]. Therefore, this review explores the established pathways of MPs transfer and the global prevalence of MPs in tuna, focusing on how these pollutants enter the seafood supply chain and the toxicological implications for aquatic species and human health. A comprehensive understanding of the pathways and associated risks is necessary for the development of effective strategies to mitigate the effects of MPs on food safety and public health.

## 2. Search Strategy

A comprehensive literature search was performed to identify original studies examining the occurrence of MPs in tuna species. The search covered publications from 1 January 2000 to 26 February 2025, using three major databases: Scopus, Web of Science, and PubMed. A total of 277 records were retrieved and imported into EndNote for reference management. After removing duplicates, 154 unique articles remained and were screened based on titles and abstracts. Out of these, 35 articles were selected for full-text review. Following a comprehensive assessment, a total of 19 studies were identified as meeting the eligibility criteria and were subsequently included for data extraction.

**Figure 1:**
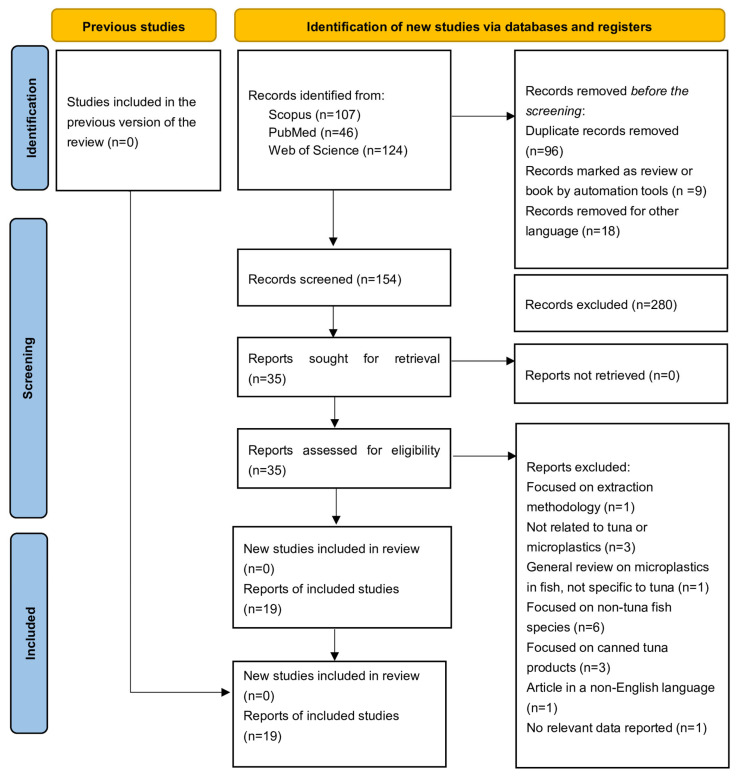
Schematic of the selection process of articles based on PRISMA.

The search strategy combined a wide range of keywords related to MPs and plastic-related pollutants, using terms including, (polystyrene OR “plastic microparticle” OR mesoplastic OR “plastic nanoparticle” OR “micro-sized plastic” OR “microscopic plastic” OR “plastic microfiber” OR “plastic fragments” OR micro-debris OR “micro debris” OR “synthetic polymer” OR “synthetic particle” OR “microplastic fiber” OR “microplastic fragment” OR “microplastic film” OR microbead OR Nylon-6 OR Polyethylene OR polypropylene OR polyacrylamide OR plexiglas OR polyamide OR “polymethyl methacrylate” OR “dimethyl phthalate” OR “diethyl phthalate” OR “di-isobutyl phthalate” OR “di-n-butyl phthalate” OR terephthalate) and (Tuna OR “bluefin tuna” OR “yellowfin tuna” OR “bigeye tuna” OR “tuna fish” OR thunnus OR “marine tuna” OR “pelagic tuna” OR “atlantic tuna” OR “tropical tuna” OR “tuna species” OR “thunnus thynnus” OR “thunnus albacares” OR “thunnus obesus” OR “thunnus maccoyii” OR “thunnus orientalis” OR “thunnus atlanticus” OR “thunnus tonggol” OR “katsuwonus pelamis” OR “skipjack tuna” OR “albacore tuna” OR “southern tuna” OR “pacific tuna” OR “blackfin tuna” OR “longtail tuna”). All search steps were completed using the PRISMA guideline, as demonstrated in Figure 1. Studies were included if they were original research articles, published in English, reported sufficient and extractable data, and focused exclusively on tuna species. Exclusion criteria included studies on species other than tuna, review articles, book chapters, conference abstracts, non-English publications, and studies with insufficient or unclear data. This systematic approach included only relevant, high-quality studies in the final review.

## 3. General Pathways of Microplastics into the Human Food Chain

MPs enter the food chain through multiple pathways, primarily through aquatic, terrestrial, and atmospheric routes. Their small size, persistence, and ability to interact with environmental factors make them highly translocatable, leading to widespread contamination in food sources [29], particularly seafood [30,31,32], drinking water [33,34], salt [35,36], honey [37], milk [38,39], and beer [40].

### 3.1. Aquatic Pathway

MPs enter aquatic ecosystems through multiple pathways, with rivers transporting 70–80% of plastics to oceans [41], leading to extensive deposition of over 0.25 Mt of floating plastic pieces and an annual influx of 11 Mt of plastics into marine environments [42]. The lack of proper waste management exacerbates this problem, with projections indicating 23 to 37 Mt of plastic waste could enter oceans annually by 2040 [43,44]. The largest contributors to MPs pollution in the oceans are China, Indonesia, Philippines, Vietnam, and Sri Lanka, respectively [45,46]. Once in aquatic environments, plastic debris from industrial, agricultural and domestic sources accumulates and breaks down into MPs through mechanical, chemical and biological weathering [47]. For example, a disposable polystyrene (PS) coffee cup lid degrades within 56 days, releasing MPs at a rate of 1.26 × 10^8^ particles per ML [48].

Microbeads and microfibers, common sources of MPs, have persisted in marine ecosystems for decades and are frequently mistaken for food by benthic organisms such as corals, sea urchins, and zooplankton [11,49]. MPs are consumed by various marine species at different trophic levels, including zooplankton [50], fish [51,52], crab [53], shrimp [54,55], snail [56] and lobster [57]. This trophic transfer results in bioaccumulation, with MPs concentrating in the gastrointestinal tracts and tissues of fish and marine mammals due to longer retention times compared to excretion rates [58,59]. Studies on species such as zebrafish reveal that waterborne exposure leads to significantly higher MPs concentrations in tissues than foodborne exposure, though trophic transfer remains a critical pathway for apex predators [60,61]. MPs can disrupt growth, reproduction, and digestive functions in marine organisms, raising concerns about ecosystem health [62].

Humans are primarily exposed to MPs via seafood consumption, with contamination levels varying by species, region, and processing methods [63]. Mollusks from Asian coasts show the highest levels of contamination globally, with bivalves containing 2.1–10.5 MPs/g [64] and cultured oysters 0.62 MPs/g [65]. Fish from Chinese coastal regions, such as those of Dongshan Bay and Beibu Gulf, contain 0.027–1.98 MPs per individual [66,67]. In the U.S., breaded shrimp products contain up to 370 ± 580 MPs per serving, contributing to an estimated annual adult exposure of 11,000 ± 29,000 MPs through proteins, including seafood, meat, and plant-based products [68]. Australian studies report lower MPs abundance in wild-caught fish (0.94 MPs/individual) [69] but higher levels in store-bought barramundi (0.02–0.19 MPs/g) [1], with sardines showing the highest plastic concentration (0.3 mg/g tissue) among tested seafood [70]. Fibers less than 1.5 mm, predominantly composed of polyester (PES), PE, and polyethylene terephthalate (PET), are the most common types of MPs [65]. Non-edible tissues such as gills and digestive systems generally contain more MPs than edible flesh, though minor translocation to muscle tissues may occur naturally in living organisms [64,65,66].

### 3.2. Terrestrial Pathway

MPs contamination in soils can result from the use of plastic mulching, the application of sewage sludge as fertilizer, the irrigation with contaminated water, and the atmospheric deposition of airborne MPs [71,72]. Soil characteristics, cultivation practices, and the diversity of soil biota influence the fate and dispersion of MPs in the soil [73]. MPs deposited on the soil are readily absorbed by plants, either directly through their roots or indirectly through damage caused by herbivores and mechanical injuries [74].

Plastic mulching is identified as a major contributor to both macroplastic and microplastic contamination in soils, with concentrations increasing over time in fields under continuous mulching [75]. MPs can negatively impact the soil biota, such as earthworms and nematodes, and potentially accumulate in the soil [76]. MPs can potentially damage crop plants by accumulating near roots and hindering water and nutrient uptake as MPs enter the terrestrial food chain, have been detected in livestock excreta, earthworms, and crop plants, raising concerns about their impact on agricultural ecosystems and human health [77]. In Italy, the contamination levels differ in various products, with apples showing the highest concentration among fruits and carrots among vegetables. The analysis indicated that the smallest MP size was detected in carrot samples, measuring 1.51 μm, whereas the largest size was found in lettuce, measuring 2.52 μm [78]. In Türkiye, the analysis revealed that the maximum average concentration of MPs in tomato samples was 3.63 ± 1.39 particles/g. Tomatoes also represented the highest estimated annual intake of MPs at 398,520 particles per individual per year and an estimated daily intake for children at 68.24 particles kg/day. Fruits generally showed higher levels of MPs contamination of less than 10 μm compared to vegetables [79].

This terrestrial accumulation is a significant concern not only for land-based ecosystems but also for aquatic ones, including marine environments such as those inhabited by tuna. This is because MPs from agricultural soils are easily mobilized through surface runoff, leaching into groundwater, and drainage systems and eventually flowing into rivers and oceans [80]. For instance, research in agricultural catchments has shown that rainfall events effectively wash MPs from mulched fields into adjacent drainage ditches and streams, ultimately carrying them into larger river systems [81,82]. A study demonstrated this direct hydrological transport, finding increased concentrations of agricultural-derived plastics in river sediments downstream of intensively farmed areas [83]. Therefore, land practices directly contribute to the increasing burden of plastic pollution in the seas, impacting marine food and potentially larger fish species. Furthermore, when animals ingest contaminated plants, MPs can move through the terrestrial food chain, entering the diets of omnivores and humans. The potential impact of MPs on agricultural productivity, soil health, and food safety is an area of ongoing research [72].

### 3.3. Atmospheric Pathway

The atmospheric transport and deposition of MPs represent a crucial, yet often overlooked, pathway for these contaminants to reach even the most remote aquatic and marine ecosystems, ultimately impacting marine food and species like tuna [84,85]. In London, deposition rates of 575–1008 MPs/m^2^/day were observed, predominantly fibrous in nature [86]. Similarly, Paris experienced the fallout of 2–355 particles/m^2^/day, with higher rates in urban areas [87]. These MPs can travel long distances, reach remote locations, and settle in terrestrial ecosystems, water bodies, and food crops [88]. The particles are primarily smaller than 100 μm, with amorphous fragments dominating the fibers in some cases. Atmospheric MPs pose potential health risks, with annual ingestion estimates of 1.9 × 10^5^ to 1.3 × 10^6^ particles through deposition in food and drinks, comparable to inhalation exposure [89]. Airborne MPs are particularly concerned in urban environments where high levels of plastic waste and industrial activities are prevalent. Sources of airborne MPs include textiles, traffic-related particles, and waste incineration, distribution, and fate of airborne MPs are influenced by meteorological conditions, urban topography, and particle characteristics [90,91]. Meteorological factors such as wind patterns, precipitation, humidity, and temperature inversions affect airborne MPs transport, deposition, and atmospheric residence time. For example, strong winds disperse airborne MPs over long distances, while rainfall removes them through wet deposition [90]. This wet deposition is particularly relevant for aquatic systems, since rainfall directly washes airborne MPs from the atmosphere into surface waters, making them available for ingestion by marine organisms, from plankton to fish [85]. Urban topography plays a role in airborne MPs accumulation, with dense building structures creating stagnant air that traps particles, while vegetation can act as a natural filter to reduce airborne MPs concentrations [92]. Traffic corridors generate turbulence that disperses or deposits airborne MPs locally [93]. Particle characteristics, such as size, density, polymer type, and hydrophobicity, also determine their behavior; smaller particles remain airborne longer, while larger ones settle faster [94]. Continuous input of atmospherically deposited MPs means that marine ecosystems are constantly exposed to this form of pollution, contributing to the overall MPs load in the oceans and the subsequent potential for bioaccumulation in marine food [95].

### 3.4. Food Packaging and Processing

This section details how MPs originating from food packaging and processing represent a significant source that contributes to the overall environmental burden, ultimately leading to the presence of these contaminants in marine ecosystems and species such as tuna.

MPs can enter the food chain through direct contamination during food packaging, processing, and storage. The widespread use of plastic packaging materials, estimated at 146 Mt in 2015, has raised concerns about MPs transfer into food products [96]. Many food items, including canned goods, beverages, frozen meals, and even tea brewed with plastic tea bags, have been found to contain MPs [10,97]. Plastic tea bags can release up to 11.6 billion MPs and 3.1 billion nanoplastics into a single cup of tea at 95 °C [20]. While plastic packaging offers benefits such as reducing food waste by extending shelf life, lowering transportation costs due to its lightweight nature, and improving food safety, it also poses risks of MPs contamination [98]. MPs can leach plastic packaging materials such as polyethylene into food products under certain conditions, such as heat exposure or prolonged contact [99]. UV radiation effectively initiates photo degradation by breaking down polymer chains through photochemical reactions. This process generates crucial intermediates, including free radicals and carbonyl compounds, which lead to the formation of smaller molecular structures and MPs [100]. Elevated temperatures facilitate thermal degradation through mechanisms such as thermal oxidation and pyrolysis, which cause products including alkenes, alkanes, and aromatic hydrocarbons. Mechanical stress results in physical fragmentation, occurring without the formation of specific chemical intermediates, while simultaneously increasing the surface area available for subsequent degradation. Additionally, microbial degradation involves enzymatic processes that generate by-products such as monomers and organic acids [101]. These processes result in the release of MPs from packaging materials into food products through mechanisms such as abrasion, surface erosion, and leaching. Additionally, UV radiation and elevated temperatures exacerbate these phenomena by enhancing fragmentation and facilitating the release of MPs [10]. Consumer waste from these packaged foods, including the packaging itself and any food residue containing MPs, frequently enters municipal waste streams [102]. Inadequate waste management then allows a significant portion of these plastics, either as larger items or already fragmented MPs, to escape into the environment [103]. This represents a direct pathway for land-based plastic pollution to reach aquatic ecosystems via runoff, wastewater discharge, and wind transport, ultimately contributing to the oceanic MPs burden where marine life, including tuna, is exposed [80]. During food production, plastic packaging can unintentionally introduce MPs into food. Polyolefin, which includes PE in its various forms (LDPE, LLDPE, HDPE) and PP, plays a leading role in the packaging industry due to its remarkable versatility, durability, and exceptional moisture and gas barrier properties [104,105]. However, their environmental persistence has raised concerns since only a small fraction is recycled, while most end up in landfills or as litter [106].

## 4. Toxicological and Health Risks of Microplastics for Human

**Figure 2:**
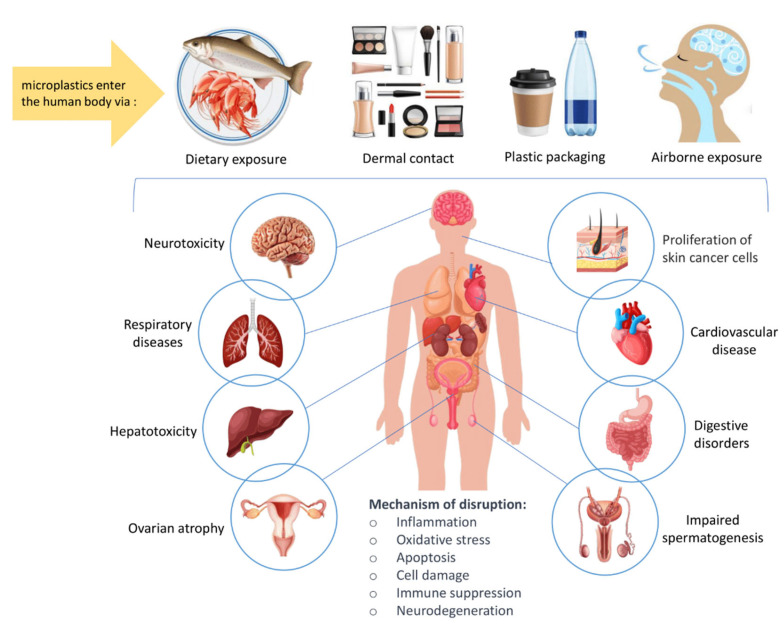
Exposure routes and the possible health effects of microplastics on human health.

Recent studies show that MPs from seafood can accumulate in fish muscle and digestive organs, allowing them to enter the human body. As shown in this study, MPs are transferred to the human body through the consumption of tuna fish, in which different types of polymers are detected. Beyond being a physical contaminant alone, MPs also carry chemical and biological risks due to their capacity to adsorb heavy metals, persistent organic pollutants (POPs) and pathogenic microorganisms on their surfaces. This situation requires a more comprehensive evaluation of MPs integrated into the marine food chain in terms of their possible neurotoxic, endocrine-disrupting and immunosuppressive effects on human health (Figure 2).

Potential health risks associated with MPs have attracted increasing attention, as research uncovers the widespread presence of these pollutants in the environment, particularly in food matrices. Given their small size, persistence, and the variety of chemical additives they contain, MPs pose several health risks to humans, ranging from physical damage to possible long-term toxicological effects [107].

MPs represent a significant threat to human health, as they can be absorbed through various pathways, including ingestion, inhalation, and skin contact. While the excretory system eliminates >90% of MPs ingested via feces, retained particles accumulate in tissues such as lungs, blood, placenta, and liver, raising concerns about long-term toxicity influenced by polymer type, size, shape, hydrophobicity, surface chemistry, and associated pollutants [63,108]. MPs have been detected in human feces, lungs, placenta, and blood [109] with studies linking them to skin cancer proliferation by internalizing into squamous cell carcinoma cells, increasing mitochondrial reactive oxygen species (ROS), and inflammasome pathways [110]. Reproductive health is particularly vulnerable, as MPs disrupt the hypothalamic pituitary gonadal axis, impairing spermatogenesis in males (via blood–testis barrier compromise) and causing placental dysfunction, ovarian atrophy, and endometrial issues in females [111]. In men, MPs cause testicular anomalies, alter spermatogenesis, and compromise sperm health. Females experience ovarian atrophy, uterine deformities, and placental dysfunction [112,113,114]. Both sexes suffer from endocrine imbalances, oxidative stress, and inflammation [112,115]. Animal studies confirm reduced fertility, ovarian capacity, and sperm motility due to oxidative stress, apoptosis, and inflammation [114,116]. Cardiovascular risks are also significant: MPs in carotid artery plaques correlate with higher risks of myocardial infarction, stroke, and death [117], likely driven by oxidative stress, mitochondrial dysfunction, vascular inflammation, and thrombosis [118]. Liver damage occurs through disruption of the gut–liver axis, altered microbiota, compromise of the intestinal barrier, hepatotoxicity, and possible contributions to nonalcoholic fatty liver disease [119,120]. Respiratory exposure to airborne MPs estimated at up to 3000 particles daily [121] triggers lung injury, fibrosis, and inflammation, with smaller particles causing more severe damage. In addition, MPs ingestion can cause direct physical damage to the gastrointestinal system [122]. Studies have shown that MPs can irritate and inflame the digestive tract, leading to conditions such as gut dysbiosis and increased intestinal permeability. This disruption of the gut lining allows harmful pathogens and toxins to enter the bloodstream, triggering systemic inflammation and increased susceptibility to infections. Furthermore, MPs can bioaccumulate in the gastrointestinal tract, potentially causing physical blockages, discomfort, reduced nutrient absorption, and other digestive issues [123,124]. These effects, while primarily observed in animal models, raise concerns about similar impacts on human health, particularly in populations that consume large amounts of seafood or contaminated water [125]. MPs also interfere with digestive processes and can reduce lipid digestion in simulated human gastrointestinal systems [126], with PS MPs exhibiting the highest inhibition. This reduction occurs through two mechanisms: decreased bioavailability of lipid droplets due to heteroaggregation and reduced lipase activity caused by adsorption and structural changes [126]. Although only particles up to 5 µm can significantly cross epithelial barriers, larger MPs can still affect human health by affecting the epithelial barrier and the lumen of the gastrointestinal and respiratory tracts. Potential adverse effects include chemical leaching, sorption, and desorption of contaminants, alteration of nutritional status, and dysbiosis [127]. Additionally, MPs can disrupt gut homeostasis by altering microbial communities and interacting with bacterial enzymes, which may negatively impact microbial functions essential for health outcomes [128]. The gastrointestinal tract serves as both an entry point and a barrier for MPs, which can undergo physicochemical transformations during digestion [129].

Beyond the GI system, MPs have been shown to cross biological barriers and accumulate in other tissues such as the brain. They can infiltrate brain tissue via systemic circulation or retrograde transport through olfactory nerve endings. This accumulation may cause neurotoxicity through oxidative stress, inflammation, and neurotransmitter imbalances. Studies indicate that MP exposure can impair neurotransmitter systems, inhibit acetylcholinesterase activity, and lead to cognitive dysfunctions such as memory deficits and behavioral abnormalities in both fish and humans [130,131]. MPs are also involved in disrupting the gut–brain axis by promoting inflammation and oxidative stress in the gut microbiota. Such disruptions may contribute to neurodegenerative diseases like Alzheimer’s and Parkinson’s [132]. Moreover, endocrine-disrupting chemicals carried by MPs further exacerbate health risks. These chemicals are linked to cognitive dysfunction, developmental delays in children, and an increased risk of neurodegeneration in adults. Chronic exposure to MPs has systemic implications due to their ability to alter metabolic pathways in tissues such as the liver and kidney. For example, research has shown that MPs can migrate from the gut into these organs, causing tissue-level inflammation and metabolic dysfunctions [133].

## 5. Ecotoxicological Risks of Microplastics in Aquatic Organisms

**Figure 3:**
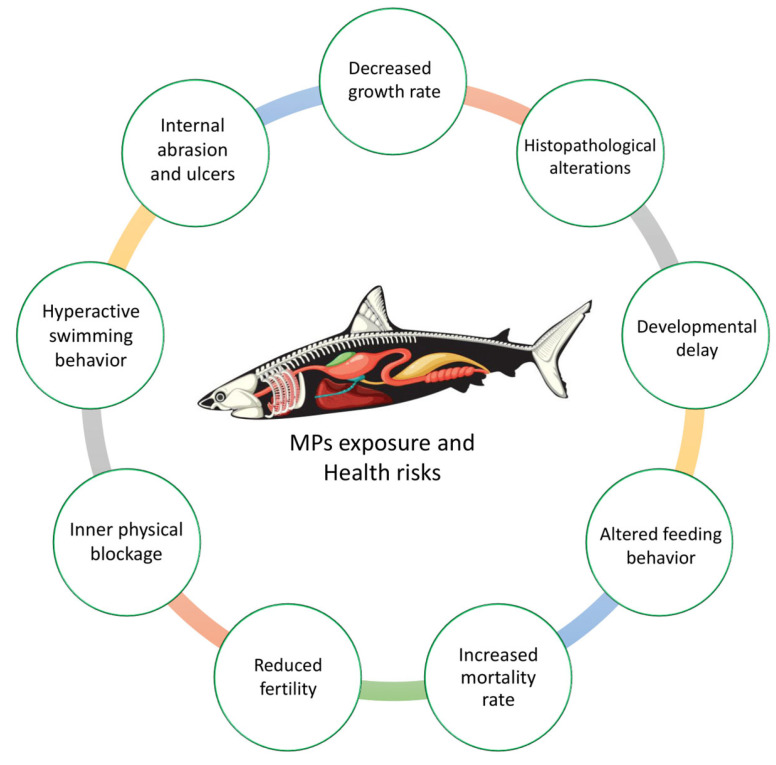
Toxicological risks of microplastics in aquatic organisms.

MPs pose significant environmental and toxicological threats to aquatic ecosystems (Figure 3), with ingestion by marine organisms leading to harm and facilitating the transfer of concentrated environmental contaminants through food chains [134]. These particles disrupt nutrient cycling and cause stress in organisms, threatening ecosystem composition and stability [34]. Ecotoxicological studies demonstrate that MPs significantly negatively impact ingestion and reproduction in aquatic species by disrupting energy metabolism and stress defense mechanisms [135]. Plastic additives, including plasticizers, flame retardants, and stabilizers, contribute to the intensification of these effects by modifying biological processes. These additives induce developmental malformations, reproductive toxicity, hepatotoxicity, neurotoxicity, and cardiovascular disorders in aquatic organisms through the disruption of cellular mechanisms [17,135].

Physiological stress caused by MPs manifests itself as behavioral alterations, immune responses, and metabolic changes in marine life. The alteration of neurotransmitter biomarkers through exposure to water and food further underscores their neurotoxic potential [59]. MPs not only impair individual organisms but also disrupt broader ecosystem functions, including nutrient cycling and trophic network dynamics, thereby threatening biodiversity and ecological balance [34]. Despite these findings, significant uncertainty remains due to the preliminary stage of research in this field. Current studies suggest that the physiological consequences of exposure to MPs are likely more extensive than currently documented. Researchers emphasize the need for additional studies to uncover the full scope of pathologies caused by different exposure routes, particularly the long-term effects of chronic, low-dose exposure [48]. Longitudinal studies are critical to understanding cumulative impacts on reproduction, immune function, and genetic expression in aquatic species.

### 5.1. Ingestion and Accumulation

MPs are easily ingested by low-trophic fauna due to their small size, which poses significant risks to organism health. Filter-feeding species, such as mollusks, copepods, and certain fish, are particularly susceptible to MP ingestion [16]. A recent study found that 11% of studied mesopelagic fish contained MPs, suggesting a striped dolphin (*Stenella coeruleoalba*) could ingest around 463 million MPs from contaminated prey [136]. Ingestion of MPs can have various negative effects on aquatic organisms [16], including reduced consumption of natural prey, decreased growth rates, histopathological alterations, developmental delays, altered feeding behavior [137], internal abrasions, ulcers, and physical blockages in the gastrointestinal tract [138,139]. These impacts can impair digestion and nutrient absorption, reduce reproductive fitness, and increase mortality rates due to diminished predator avoidance [140]. Although MP accumulation in the gastrointestinal tract may be temporary, lifetime exposure is likely high and increasing [138]. These effects have broader implications for marine trophic networks and ecosystem stability.

MPs may accumulate in marine organisms, leading to bioaccumulation in tissues such as the liver and circulatory system over time. MPs increase in concentration at higher trophic levels through biomagnification [141]. This includes marine mammals and humans as the main predators that consume seafood contaminated with MPs. However, bioaccumulation modeling studies suggest that the biomagnification potential of MPs within cetacean trophic networks is limited compared to POPs [142]. Existing field evidence does not robustly support biomagnification in generalized marine; instead, bioaccumulation within trophic levels appears to be more prominent than biomagnification at higher trophic levels. While bioaccumulation through water exposure is evident, the biomagnification pathway remains debated. Overall findings suggest that while MPs bioaccumulation occurs within trophic levels, there is no clear evidence of biomagnification at higher trophic levels in situ [143]. This debate emphasizes the need for more research on the long-term effects of MPs and their accumulation in ecosystems.

### 5.2. Chemical Leaching and Toxic Additives

MPs are a rising concern due to hazardous additives in plastics, including phthalates, bisphenol A, and heavy metals. These additives can leach out as plastics degrade, contaminating surrounding water and ecosystems [144,145]. The release of these chemicals is influenced by factors such as particle size, light exposure, temperature, and water conditions [145]. Upon release, these chemicals can adversely affect aquatic organisms, leading to significant concerns such as endocrine disruption, reproductive toxicity, and neurotoxicity [133]. Ecotoxicological studies have shown that MPs and its additives disrupt biological processes related to energy metabolism, stress response, and cytoskeletal dynamics in aquatic organisms [146]. Furthermore, these contaminants can be transferred along the food chain, potentially affecting higher trophic levels and human health [135,147]. MPs particles have a large surface area to volume ratio, which enhances their adherence potential for environmental contaminants such as POPs [15]. These pollutants, including polychlorinated biphenyls (PCBs) and polycyclic aromatic hydrocarbons (PAHs), concentrate on the surfaces of MPs [148]. For example, research on beach-deposited MPs revealed PCB concentrations up to 2750 ng/g and PAH concentrations up to 24,000 ng/g [21]. The sorption of POPs to MPs is influenced by environmental factors such as pH, salinity, and temperature of seawater, as well as MPs‘ properties such as polymer type, color, and size [149].

### 5.3. Impact on Reproductive Health and Development

MPs and nanoplastics pose significant threats to reproductive health in ecosystems. Exposure to MPs has been shown to alter reproductive systems, leading to altered gonadal development (hypothalamic-pituitary gonadal axis), disrupting the neuroendocrine system, reducing fertility, and changing sex ratios in some species. For example, in marine fish species, exposure to MPs has been associated with reduced sperm motility and impaired egg quality, ultimately affecting reproduction [114,115,150]. In addition to reproductive toxicity, MPs can affect the development of aquatic organisms. Research has indicated that the early life stages of both fish and invertebrates are particularly susceptible to exposure to MPs. These organisms may absorb MPs through their gills or skin, thereby increasing their vulnerability to potential adverse effects. This can lead to developmental delays, impaired organ formation, and increased mortality rates [151]. MPs also affect offspring development, leading to increased mortality rates, metabolic perturbations, and weak immunity in rodents. In aquatic species, MPs reduce reproduction rates and cause abnormal larval development [112]. The prevalence of MPs presents significant challenges to both biodiversity and reproductive health. This situation underscores the urgent need for comprehensive research and targeted interventions to address and mitigate their impacts effectively.

### 5.4. Behavioral Changes and Ecosystem Disruption

Exposure to MPs leads to abnormal behaviors in fish, such as hyperactive swimming patterns, reduced predator avoidance, and decreased foraging efficiency, which affect survival and reproductive success, ultimately contributing to population declines. These particles also accumulate in primary producers like plankton, disrupting energy flow through trophic networks as contaminated zooplankton become less nutritious for higher trophic levels, threatening the health of predators and ecosystem stability [152]. Additionally, MPs act as carriers for toxic pollutants, including heavy metals and organic chemicals, which bioaccumulate in organisms, causing organ damage, oxidative stress, and reproductive decline [153]. The combined effects of behavioral alterations, chemical toxicity, and energy flow disruptions compromise individual fitness, weaken predator-prey dynamics, and destabilize aquatic ecosystems, highlighting the urgent need for mitigation strategies to address this pervasive environmental threat [59].

## 6. Prevalence of Microplastics in Tuna Fish

Tuna, a large pelagic predator fish belonging to the Scombridae family [154], is highly migratory and holds significant value in global marine fisheries. It is widely consumed and commercially important for both national and international markets, with an annual catch of 0.2 Mt [155]. Despite its nutritional benefits, there is growing concern about contaminants in tuna. As the main predators, tuna are exposed to and accumulate chemicals throughout the trophic chain [156,157].

In regions where fish constitute a significant portion of the local diet, high levels of MP contamination not only pose severe health risks but also serve as indicators of pollution [158]. In several studies, fish have been used as natural indicators for the assessment of water pollution, with particular emphasis on their critical role in monitoring MPs within aquatic ecosystems [158,159]. The effectiveness of fish as indicators of pollution is influenced by several factors, including the concentration of MPs present in the water, the biological characteristics of the fish, the duration of their exposure, environmental conditions, and their age, size, and habitat. Despite extensive research on MPs pollution conducted globally, there remains a significant gap in knowledge regarding MPs contamination in commercially important predatory species such as tuna. Tuna species serve as apex predators within oceanic ecosystems and are known for their long-distance migratory patterns. They play a crucial role in clarifying the processes of accumulation and potential bioaccumulation of MPs within marine food [160]. The elevated incidence of MPs detection in tuna may be attributed to their feeding behavior, which occurs both diurnally and nocturnally, thus increasing the likelihood of ingesting these particles. Tuna species show vertical migration between deep and surface waters over a 24 h cycle, which significantly increases their likelihood of exposure to MPs [161]. Tuna are subjected to MPs exposure through two primary mechanisms: respiration and opportunistic feeding. By swimming with their mouths open, they facilitate the flow of water over their gills to obtain oxygen and food. Given that tuna are not particularly strong swimmers in terms of directed movement, their probability of consuming MPs is closely associated with their habitat and the specific prey they target. It is assumed that their primary prey varies according to their location and that their stomach contents are indicative of the diversity of prey species present in different marine regions [162]. Predatory tuna species are characterized by their consumption of relatively large prey items, such as fish and cephalopods, reflecting a selective feeding strategy. Considering this feeding behavior, as well as the small size of MPs identified within their gastrointestinal tracts, it is improbable that these species confuse plastic debris with actual prey. Instead, the presence of MPs is more likely attributable to secondary ingestion through contaminated prey or incidental intake during typical feeding activities. This is particularly significant in instances where larger plastic fragments, including threads measuring up to 11 cm, have been documented [24]. Furthermore, these indicators are essential for understanding the mechanisms by which humans may be exposed to MPs through the consumption of fish and seafood, thus highlighting significant public health concerns [163].

### 6.1. Evaluation of Microplastics Frequency, Characteristics, and Concentrations in Tuna Species

**Figure 4:**
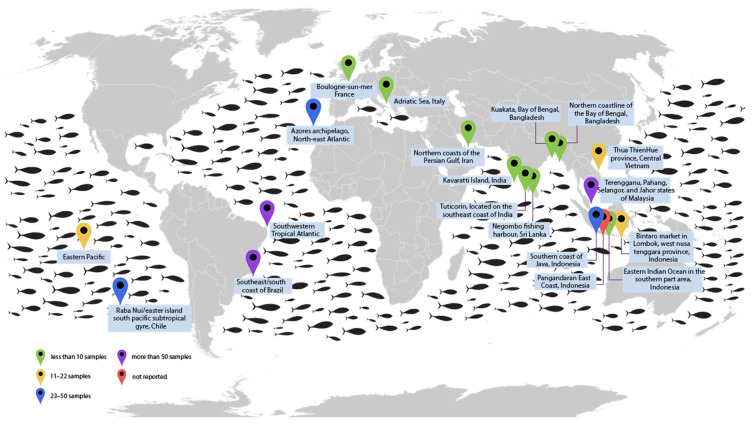
Global distribution map of tuna species and the sampling locations investigated for microplastics contamination based on current systematic review.

Detection of MPs in seafood has become a critical issue for human health, as well as marine ecosystems. Studies have found MPs in *Thunnus tonggol*, *Katsuwonus pelamis*, *Euthynnus affinis*, *Auxis rochei*, and other tuna species. They are commonly found in the Atlantic, Pacific, and Indian Oceans, as well as in semi-enclosed seas such as the Mediterranean and the Adriatic Sea. The global distribution of tuna species and the sampling locations investigated for MPs contamination to date are illustrated in Figure 3. As shown in Figure 4, studies have been conducted across all three major oceans: the Atlantic, Pacific, and Indian. However, the majority of studies have been focused on the Asian continent, while no published studies are available from certain regions such as North America, Africa, and Australia.

Data from 19 different studies included in the systematic review revealed that MPs detected in different tuna species showed significant diversity in terms of density, type, physical structure, and distribution to organs, as detailed in Table 1. In the following, this diversity is comparatively evaluated on the basis of species and MPs properties (polymer type, size, shape, and color) and concentration values.

**Figure 5:**
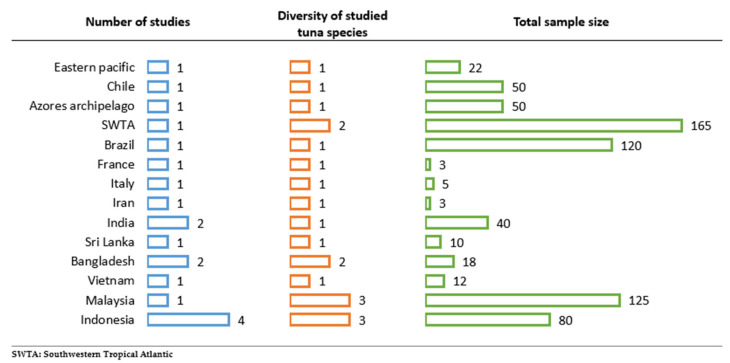
Geographical distribution of study locations, tuna species diversity, and sample size for microplastic contamination analysis.

Countries and regions where studies have been conducted, along with the diversity of tuna species investigated in each area and the total number of samples collected, are presented in Figure 5. Most of the studies were conducted in Indonesia, where three tuna species were investigated, with a total of 80 samples analyzed.

The species most studied is *Katsuwonus pelamis*, and a total of 252 individuals were analyzed in 6 different geographies. This provides us with the opportunity to comparatively examine how individuals of the same species respond to different environmental pressures. The MP concentration detected in this species is quite variable. For example, while 1.42 ± 0.29 MPs/g was detected in samples collected from the Negombo port of Sri Lanka [166], very high values such as 7.16 ± 1.36 MPs/g in muscle tissue, 2.16 ± 0.26 MPs/g in intestine, and 2.66 ± 0.40 MPs/g in the gills were reported in Kuakata samples from Bangladesh [22]. This value was determined as 1.65 ± 1.2 MPs/individual in samples of the same species from the Brazilian coast [168]. *Katsuwonus pelamis* and *Thunnus obesus* species are remarkable examples in terms of both individual load and inter-tissue spread. *Thunnus obesus* is particularly notable for its high MPs load on the coasts of the Bay of Bengal in Bangladesh. The total MPs per individual in this species are quite high at 42.13 ± 13.58 MPs/individual. MPs detection in both the muscle, gill, and intestine of *Thunnus obesus* reflects high MPs exposure related to the feeding and filtration structure of this species [161]. While Pacific samples of *Thunnus albacares* contain low levels of particles, high MPs were reported in Atlantic samples (10.33 ± 14.06 MPs/individual) [7]. In the Bay of Bengal, high concentrations of MPs were detected in *Thunnus obesus* (42.13 ± 13.58 MPs/individual), indicating that this region is heavily contaminated and underscores the widespread nature of MPs pollution in marine environments. In contrast, lower but still significant levels of MPs were found in *Thunnus thynnus* from the Adriatic Sea (0.16 to 0.27 MPs/g), demonstrating that MPs contamination is not confined to tropical regions and affects diverse marine ecosystems globally. *Katsuwonus pelamis* consistently shows high levels of MPs across different locations, indicating a high susceptibility to MPs ingestion. *Thunnus albacares* and *Thunnus thynnus* show moderate to high levels, suggesting that larger species may also be significantly affected.

The morphological features of MPs are also instructive in terms of predicting contamination sources. For example, the most common form in many studies is fiber, which suggests that a significant portion of MPs originate from synthetic textile products and fishing equipment (e.g., fishing lines, nets, and ropes). Fiber structures can usually mix with water during laundry and pass through wastewater treatment plants and reach the marine environment. In addition, MPs in the form of fragments are formed by the breakdown of larger plastic materials in the environment as a result of physical, chemical, or biological processes. Other forms such as film, foam, and pellet can be associated with packaging waste, PS foam products, and industrial production raw materials, respectively.

The detected MPs size range also provides important data on the nature of the sources. MPs sizes vary by species and study. For example, while smaller particles such as 23–75 µm are dominant in *Thunnus tonggol*, the average size was measured as 0.77 ± 0.92 mm in *Thunnus albacares*. Microsized particles such as <10 µm were detected in *Thunnus thynnus* samples, raising concerns that these particles may leak beyond the digestive system into the circulation and muscle tissue. This risk is even more striking in cases where samples are detected densely in muscle tissues (e.g., *Katsuwonus pelamis* and *Thunnus alalunga*). MPs, which are generally concentrated in the <5 mm range in studies; especially the dominance of particles <1 mm in size, indicate that environmental exposure is long-term and widespread. The dominant size range is 0.5–2.5 mm, indicating that small to medium-sized MPs are more likely to be ingested by tuna species. Particles of these sizes increase both the risk of bioaccumulation at trophic levels and have permanent effects on organisms because they are not biodigestible.

When the color distribution of MPs detected in the studies is reviewed, it is observed that tones such as black, blue, transparent, red, and white are dominant. Colored particles have been reported in a wider range, especially in the species *Auxis rochei*, *Katsuwonus pelamis* and *Thunnus tonggol*. These colors are generally widely used in products based on human activities (e.g., textile dyes, net yarns, and packaging materials). In addition, the possibility of colored MPs being ingested as food increases, which increases the biological risks. The findings in Table 1 clearly demonstrate that MPs contamination in tuna species is widespread, complex, and multi-sourced. The variety of polymers and the range of shapes and colors indicate that MPs are not limited to coastal pollution but also circulate at an oceanic scale. However, the detection of high levels of MPs, even in muscle tissue, poses a serious public health risk due to the prevalence of these organisms in human consumption.

### 6.2. Relationship Between Regional Distribution of Microplastics Concentrations and Properties and Local Anthropogenic Factors

Furthermore, the density and characteristic features of the MPs detected in the tuna were evaluated in relation to the demographic, industrial, and environmental characteristics of the sampling regions.

The levels of MPs detected in *Katsuwonus pelamis* and *Thunnus obesus* species caught on the coast of the Bay of Bengal of Bangladesh represent the highest values among all studies in terms of both the number of particles per individual and per gram. This situation can be explained by the combined effects of densely populated settlements along the coast, industrial structures based on textiles, and inadequate wastewater treatment systems. Bangladesh is a country with a particularly dense textile industry and is the world’s second largest textile exporter in ready-made garments [173]. In Bangladesh, the management of industrial wastewater still faces serious infrastructure problems. According to recent studies, a significant number of textile and dyeing plants, especially around Dhaka, lack treatment systems and their wastewater is discharged directly into rivers such as Buriganga, Turag and Sitalakhya [174]. 70 to 80% of industrial wastewater across the country has been reported to be released into natural water environments without any pretreatment [175]. This situation contributes significantly to the prevalence of MPs pollution in Bangladesh and the dominance of MPs, especially fiber-based, in aquatic environments [176]. Similarly, high levels of MPs pollution were observed in samplings conducted along the coasts of Indonesia and Malaysia [163,172]. The widespread detection of polymers used in advanced industrial processes such as EPDM, MF, PBDE, and PTFE in species such as *Euthynnus affinis* and *Auxis rochei* indicates the direct or indirect impacts of the petrochemical, electronics, and automotive industries on the marine environment [45,163]. Indonesia is one of the largest producers of plastic pollution in Southeast Asia. Especially large cities such as Jakarta and Surabaya produce approximately 3.2 million tons of plastic waste per year, 50% of which reaches the sea [80]. Similarly, the economic center of fishing in Indonesian islands such as Java and Lombok may have caused plastic-based equipment such as nets, fishing lines, and ropes to dissolve and enter the sea. As of 2019, Malaysia has become one of the countries that import the most plastic waste in the world after China’s plastic waste import ban [177]. At the same time, Malaysia is the center of electronics, automotive and chemical industries [178] and it is thought that the wastes from these industrial centers are carried to the coastal ecosystem. At the same time, the intensity of deep-sea fishing activities in these regions is another factor that increases the sources of MPs.

Low to moderate MPs loads were reported in samples from *Katsuwonus pelamis* collected in Negombo Port, Sri Lanka. However, the shape of the particles detected was in the form of fibers, films, and fragments, suggesting that fishing-related sources, especially leakage from port infrastructure, are the determining factors [179]. Direct industrial pressure is low in Sri Lanka. The Sri Lankan example may be an example of secondary pollution due to maritime activities rather than direct industrial pressure.

MPs loads of 1.65 ± 1.2 MPs/individual were observed in *Katsuwonus pelamis* species on the southeast coast of Brazil. This region is under the intense influence of industrial and port cities, especially São Paulo and Rio de Janeiro. MPs detected in this region have a wide spectrum in terms of coloration, as well as polymers such as PET, PP and PU. The main sources of MPs detected in Brazilian coastal waters are household waste from urbanized coastal areas, uncontrolled use of plastic packaging, and wastewater discharges associated with textile fibers [180]. Intense population pressure and inadequate waste management, especially in metropolitan areas such as São Paulo and Rio de Janeiro, lead to an increase in particles in the form of fibers and films reaching the marine environment. Additionally, recent analyses conducted on the southern coast of Brazil reveal that a significant portion of MPs originate from fishing activities, especially the inclusion of nets, fishing lines, and ropes in the sea [181].

MPs concentrations were reported to be very low in *Thunnus alalunga* individuals sampled from the northern coast of France (Boulogne-sur-Mer). The European Union has developed pioneering policy frameworks and introduced specific plastics to combat MPs pollution [182]. France has reinforced efforts in this area by banning microbead cosmetic particles under its 2020 AGEC law, in line with EU Regulation 2023/2055 restricting personal-care MPs [183]. Furthermore, all new washing machines sold from January 2025 will need to be equipped with microfiber filters to reduce microfiber emissions from synthetic textiles [184]. France actively participates in Action 52: Zero Pellet Loss, a key measure under the OSPAR Convention Regional Action Plan on Marine Litter. This initiative aims to eliminate the unintentional release of pre-production plastic pellets throughout the entire plastic supply chain, with particular attention to the handling and transport phases [185]. These efforts, together with the EU’s Marine Strategy Framework Directive (2008/56/EC), set out a holistic policy approach to preventing, monitoring and regulating MPs [186].

On the other hand, the detection of MPs with a size of <10 µm in muscle tissue in *Thunnus thynnus* samples caught in the Mediterranean basin is related to the capacity of the Mediterranean Sea, which has the characteristics of a closed sea, to accumulate pollutants. The Mediterranean Basin is one of the marine systems with the highest MPs density in the world due to its semi-enclosed structure and intense anthropogenic pressure [187,188]. Primary sources of pollution are domestic wastewater originating from coastal urban areas, discharges from industrial facilities, tourism-related activities, maritime transport operations, and plastic debris associated with fishing gear [189]. Recent studies, especially in the western and northeast Mediterranean, reveal that MPs in the form of fibers and fragments based on PE, PP, and PES are predominant in the water column and sediments [190].

The detected MPs are directly related not only to the fish species and individual physiology, but also to the waste infrastructure, industrial density, coastal use, and environmental management capacity of the sampling region. While high pollution levels, especially in regions such as South and Southeast Asia, can be considered a natural result of incompatibility between industry and environmental policies and inadequate infrastructure, European samples exhibit relatively low pollution profiles due to effective environmental management. This clearly shows that MPs are not distributed homogeneously on a global scale; on the contrary, they are a direct result of regional human activities.

## 7. Detection and Characterization of Microplastics in Tuna Species

Several sample preparation steps have been mentioned in the existing literature for the detection of MPs in tuna species. In the first step, the target tissues of tuna species, such as the muscle, gastrointestinal tract, liver, and gills, were separated and weighed precisely. After dissection, samples undergo chemical digestion to remove organic matter and enable MPs extraction. Commonly used digestion reagents include 30% hydrogen peroxide (H_2_O_2_) and potassium hydroxide (KOH) solutions [45], which efficiently dissolve biological tissues at temperatures typically between 60 and 75 °C over 24 to 48 h [163]. In some cases, additives like Fe(II) ions are added to improve the oxidative digestion process [164]. After digestion, density separation techniques using saturated sodium chloride (NaCl) [45] or zinc chloride (ZnCl_2_) [160] solutions are used to float MPs particles, helping separate them from denser residual materials. Once digestion and density separation are done, samples are filtered through membrane filters such as glass fiber filters or nitrocellulose membranes with pore sizes from 0.3 to 1.2 μm [162]. The filters are then dried under controlled laboratory conditions.

In the tuna species MPs contamination studies, visual sorting was conducted using stereomicroscopes or digital microscopes [125], which enable the sizing, counting, and morphological classification of MPs into types such as fibers, fragments, and films [165]. Morphological definitions include fibers as thin, elongated particles with uniform thickness, fragments as irregularly shaped pieces with sharp edges, and films as thin sheets commonly derived from plastic bags [165]. Color and size distributions were also documented, often using image analysis software such as ImageJ (v.1.50i; http://imagej.nih.gov, accessed on 21 June 2025) for precise length measurements [164]. To differentiate plastics from non-plastic particulates, the hot needle test is often applied, where suspected particles deform or melt upon contact with a heated needle, confirming their plastic nature [161].

For chemical identification of MPs, advanced spectroscopic techniques were utilized. Fourier Transform Infrared Spectroscopy (FTIR) is the most prevalent method, with variants including attenuated total reflectance (ATR-FTIR) and micro-FTIR imaging [22,24]. These methods non-destructively analyze the polymer composition by measuring the sample’s infrared absorption spectra, which are then compared against reference spectral libraries to determine polymer types [191]. Micro-FTIR enables mapping of numerous MPs simultaneously, though its detection limit prevents analysis of particles smaller than approximately 20 μm [192]. Raman micro-spectroscopy serves as a complementary technique, particularly useful for overcoming challenges posed by small particle sizes or organic contamination affecting FTIR spectra [193]. Raman offers higher spatial resolution (down to 1 micrometer), enabling the identification of particles below the micro-FTIR detection threshold. It can also detect morphological and crystallinity changes, especially in weathered MPs, providing additional chemical and structural information [194]. However, Raman analysis may be influenced by fluorescence and requires careful sample preparation to minimize background noise [195]. Additionally, Laser Direct Infrared (LDIR) spectroscopy offers rapid and accurate polymer identification for particles ranging from 20 to 5000 μm [196]. Surface morphology and elemental composition can also be assessed using scanning electron microscopy combined with energy-dispersive X-ray spectroscopy (SEM- JEOL model JSM 6390) equipped with EDAX [197]. SEM-EDX enables detailed visualization of particle surfaces and elemental analysis, which is essential for determining the origin and compositional heterogeneity of MPs [198].

## 8. Research Gaps and Future Directions

Current research on MPs contamination in tuna species mostly focuses on specific regions, such as the Persian Gulf, Indonesia, and the Bay of Bengal. Consequently, many other areas with significant tuna fishing activities remain underrepresented in the literature. It is essential for future studies to broaden their geographical scope to encompass a wider range of locations, thereby facilitating a more comprehensive understanding of MPs contamination in tuna on a global scale. While the prevalence of MPs in tuna is a concern, specific studies on this topic are limited, and region-specific research is required to accurately assess consumer risks. Crucially, there is also a notable research gap concerning MP dynamics in tuna species that are farmed in coastal and confined environments. Studies are needed to investigate how factors such as feed, water quality, age, size, and the specific enclosed environment influence MPs uptake, depuration (excretion), and accumulation in these farmed fish. Many studies on MPs contamination focus on specific points in time, providing a limited view of its temporal dynamics. There is a lack of information on how MP levels change over time. Therefore, longitudinal studies are crucial for understanding trends in MP contamination and for evaluating the effectiveness of pollution control measures. Environmental monitoring also needs improvement to track MP distribution and synergistic effects with other stressors. Ultimately, addressing these gaps will require interdisciplinary approaches to develop effective mitigation strategies and understand the full scope of the impacts of MPs from ocean ecosystems to human health.

## 9. Conclusions

The pervasive presence of MPs in marine environments poses a significant and escalating threat to global food safety, particularly in regard to seafood. As highlighted, seafood is a critical source of protein for billions of people worldwide, and contamination of this vital food source by plastic debris is a pressing concern. The journey of MPs from land-based sources to aquatic ecosystems and ultimately into our food chain is multifaceted, involving aquatic, terrestrial, and atmospheric pathways, along with direct contamination during food packaging and processing. Our review underscores that tuna, as an apex predator with extensive migratory patterns, is highly susceptible to accumulating MPs. This susceptibility is amplified by their feeding behaviors and diel vertical migration, increasing their exposure to MPs across various water depths. The documented presence of MPs in tuna species worldwide, as evidenced by numerous studies, raises a significant alarm given their nutritional importance and widespread consumption. The toxicological implications for aquatic organisms are already evident, ranging from physical damage and inflammation to developmental malformations, reproductive toxicity, and neurotoxicity. For human health, the potential long-term risks associated with consuming seafood contaminated with MPs, especially tuna, are still being unraveled. MPs are detected in various tissues and can leach toxic additives such as phthalates, bisphenol A, and heavy metals, contributing to endocrine disruption and other chronic health disorders. Addressing this complex environmental crisis requires a concerted global effort. Mitigation strategies must encompass improved waste management, reduced plastic production and consumption, and the development of sustainable alternatives. Developing effective regulatory frameworks, including international legally binding agreements to address plastic pollution, is critical. Continued research is vital to fully understand the long-term impacts of MP exposure on both marine ecosystems and human health, thus informing effective policies and safeguarding the future of our seafood supply.

## Figures and Tables

**Table 1:** Occurrence, characteristics, and concentrations of microplastics in tuna species as reported in various studies included in the systematic review.

Species	Common Name	Number of Samples	Sampling Location	Polymer	Size	Shape	Color	Organ	Concentration MPs/g	Abundance MPs/Individual	Detection Method	Ref.
*Thunnus tonggol*	Longtail tuna	3	Northern coasts of the Persian Gulf, Iran	NR **	23–75 µm (dominant), 75–150 µm, 150–225 µm, 225–5000 µm *	Fiber (dominant), fragment *	Black (dominant), red, blue, green, transparent *	Total gill and gut	NR	5.71	NR	[158]
								Gill	0.02 ± 0.01	5.67 ± 1.53		
								Gut	0.03 ± 0.03	3.00 ± 2.65		
*Katsuwonus pelamis*	Skipjack tuna	10	Fishing Ports in Java Island, Indonesia	NR	NR	Fiber (dominant), fragment, film	NR	Gill, muscle and GIT	0.92 ± 0.45	16.9 ± 5.86	FTIR	[164]
*Euthynnus affinis*	Mackerel tuna	50	Southern coast of Java, Indonesia	Polybrominated diphenyl ethers (PBDE)	<0.25 mm, 0.25–0.5 mm, 0.5–1 mm (dominant), 1–5 mm	Filament (dominant), angular, round	NR	GIT	NR	4 ± 3.06	GC-Mass	[45]
*Euthynnus affinis*	Mackerel tuna	69	Terengganu, Pahang, Selangor, and Jahor states of Malaysia	Ethylene propylene diene monomer (EPDM), Melamine formaldehyde (MF), Polyamide/nylon, Polytetrafluoroethylene (PTFE), PU, Rayon	0.01–1.00 mm (dominant), 1.01–2.00 mm, 2.01–3.00 mm, 3.01–4.00 mm, 4.01–5.00 mm	Fiber, fragment	Black (dominant), blue, brown, green, orange, pink, red, transparent, white, yellow	GIT, gill and muscle	0.68 ± 0.05	NR	micro-FTIR	[163]
*Thunnus tonggol*	Longtail tuna	31		EPDM, MF, Polyamide/nylon, (PTFE), PU, Rayon			Black (dominant), blue, green, orange, red, transparent, white, yellow		1.22 ± 0.14	NR		
*Auxis rochei*	Bullet tuna	25		Polyamide/nylon, PET, PTFE, Rayon			Black (dominant), blue, green, orange, purple, red, transparent, white, yellow		0.48 ± 0.06	NR		
*Thunnus albacares*	Yellowfin tuna	50	Raba Nui/easter island south pacific subtropical gyre, Chile	PP	0.3 to 0.6 mm	Fragment (dominant), flake	Green, blue, white	GIT	NR	NR	ATR-FTIR and micro-FTIR	[165]
*Katsuwonus pelamis*	Skipjack tuna	10	Negombo fishing harbour, Sri Lanka	LDPE, HDPE, PS, PP, Nylon-6,6	_	Fiber (dominant), fragment, film, foam, spheres, pellets	Blue (dominant), redblack, yellow, orange, transparent	Muscle, gill, gut	1.42 ± 0.29	NR	FTIR	[166]
*Katsuwonus pelamis*	Skipjack tuna	22	Eastern pacific	PES (dominant), PET, PS, PP, Polyacrylonitrile (PAN), Polyvinyl chloride (PVC), polyethylene-polypropylene copolymer (PE-PP), PE	0.1–0.5 mm, 0.5–1 mm, 1–2.5 mm (dominant), 2.5–5 mm	Fiber (dominant), fragment, film, line	Transparent (dominant), white, blue, pink, black, yellow, green, prpule, blue, red, gray	Total gill, esophagus, muscle, intestine, stomach	NR	NR	Raman	[162]
				PES, PS, PET, PE-PP, PP	1 to 2.5 mm (dominant), 0.1–0.5 mm, 0.5–1 mm, 2.5–5 mm	Fiber (dominant), fragment	Transparent (dominant), white, blue, purple, pink, black, green	Gill	0.01 ± 0.02	NR		
				PES, PET (dominant), PS, PE, PE-PP, PVC	0.1–0.5 mm, 0.5–1 mm, 1–2.5 mm (dominant), 2.5–5 mm	Fiber (dominant), fragment, film	Transparent (dominant), white, blue, purple	Esophagus	0.04 ± 0.09	NR		
				PET (dominant), PES, PP, PS, PAN, PE	0.1–0.5 mm, 0.5–1 mm, 1–2.5 mm (dominant), 2.5–5 mm	Fiber (dominant), thread, fragment, film	Transparent (dominant), blue, white, grey, black, pink	Muscle	0.01 ± 0.01	NR		
				PES (dominant), PET, PS, PP, PVC	0.1–0.5 mm, 0.5–1 mm, 1–2.5 mm (dominant), 2.5–5 mm	Fiber (dominant), film	White (dominant), transparent, pink, yellow, blue, black, red, grey, green, purple	Intestine	0.02 ± 0.03	NR		
				PES, PP, PET (dominant), PS, PAN, PVC	1–2.5 mm (dominant), 0.1–0.5 mm, 2.5–5 mm, 5–10 mm	Fiber (dominant), fragment, film	White (dominant), transparent, blue, black, pink, red	Stomach	0.02 ± 0.05	NR		
*Thunnus thynnus*	Bluefin tuna	5	Adriatic Sea, Italy	PE, PET, PEVA, poly(ethylene-co-vinyl acetate); PES, PP (dominant), PS, PU, PVAc, PVC	<10 µm	Fragment (dominant), filament, spheroid	Blue (dominant), brown, black, yellow, green, sky blue, red, transparent	Muscle	0.16 to 0.27	NR	FTIR and Raman	[167]
*Katsuwonus pelamis*	Skipjack tuna	30	Kavaratti Island, India	PE (dominant), PP, PES, PS, PA	0.01–0.5 mm, 0.5–1 mm (dominant), 1–1.5 mm, 1.5–2 mm, 2–2.5 mm, 2.5–3 mm, 3–3.5 mm, 3.5–4 mm, 4–4.5 mm, 4.5–5 mm	Fiber (dominant), fragment, film, foam, microbead	Blue (dominant), black, red, green, white, transparent	GIT	NR	4 ± 3	ATR-FTIR and Scanning Electron Microscopy (SEM)	[160]
*Thunnus albacares*	Yellowfin tuna	102	Southwestern Tropical Atlantic	SBR (dominant), PA, PET, PE, PU, LDPE, PVC, ABS, Alkyd Varnish, PP, PS, Poly methyl methacrylate (PMMA), Poly tetrafluoroethylene (PTFE), Chlorinated Polyisoprene	0.77 ± 0.92 mm	Fiber (dominant), film, foam, fragment, pellet	White, blue, black, yellow, red	GUT	NR	10.33 ± 14.06	Laser Directed Infra-Red spectroscopy	[7]
*Thunnus obesus*	Bigeye tuna	63		SBR, PA, PET, PE	NR	Foam (dominant), fiber, pellet, fragment	NR		NR	NR		
*Thunnus alalunga*	White tuna	3	Boulogne-sur-mer, France	PET (dominant), PP, PE, PVC, PTFE, PP, Phenoxy resin, Ethylene-vinyl acetate (EVA)	0–50 µm, 50–100 µm, 100–500 µm (dominant), 500–5000 µm	Fiber (dominant), pellet, fragment	NR	Muscle	0.33 ± 0.008	NR	micro-FTIR	[125]
*Katsuwonus pelamis*	Skipjack tuna	120	Southeast-south coast of Brazil	PA (dominant), PU, PP, PS	0.001–5 mm	Fiber/line (dominant), rigid fragment, flexible fragment	Transparent (dominant), white, black, green, red, blue	GIT	NR	1.65 ± 1.2	FTIR with an IRP restige-21 SHIMADZU mass spectrophotometer	[168]
*Euthynnus affinis*	Mackerel tuna	12	Thua Thien Hue province, Central Vietnam	Rayon, PET, PA, polyacrylic	100 μm (dominant), 100–200 μm, 200–500 μm	Fiber (dominant), fragment	White transparent (dominant), yellow-orange, blue-green, red-pink, black-grey	Fish tissue	1.0 ± 0.4	NR	FTIR-ATR	[169]
*Auxis rochei*	Bullet tuna	20	Bintaro market in Lombok, west nusa tenggara province, Indonesia	PA	100–500 µm (dominant), 500–1000 µm, >1000 µm	Fiber (dominant), film, fragment, foam, pellet	Black, yellow, red, blue, green, white	Skin and muscle tissue	0.216 ± 0.87	NR	FTIR	[170]
*Thunnas obesus*	Bigeye tuna	8	Northern coastline of the Bay of Bengal, Bangladesh	EVA, nylon, PE, PP *	<0.5 mm (dominant), 0.5–1 mm, 1–5 mm	Fiber (dominant), sheet	Violet (dominant), transparent, blue, green, red, black, pink, yellow	Total muscle, GIT, Gill	NR	42.13 ± 13.58	FTIR	[161]
								Muscle	0.375 ± 0.17	NR		
								GIT	1.37 ± 0.62	NR		
								Gill	1.57 ± 0.58	NR		
*Katsuwonus pelamis*	Skipjack tuna	50	Azores archipelago, North-East Atlantic	PS, PP, PVC, PE	0.02–1 mm	Fiber, thread, fragment (dominant)	White, blue, black (dominant)	Stomach	NR	0.08 ± 0.05	micro-FTIR	[24]
					1–5 mm				NR	0.02 ± 0.02		
					>5 mm				NR	0.06 ± 0.03		
*Katsuwonus pelamis*	Skipjack tuna	10	Tuticorin, located on the southeast coast of India	PE, PEST, PA, PS, PP, Acrylic *	500 μm to 1 mm, 1–5 mm	Fiber, fragment (equal)	Blue, transparent, green, red, black *	Total body and gut	NR	0.2 ± 0.06	FTIR and SEM-EDAX	[171]
								Body	6.67E-05 ± 0.0000001	NR		
								Gut	0.001 ± 0.0005	NR		
*Katsuwonus pelamis*	Skipjack tuna	10	Kuakata, Bay of Bengal, Bangladesh	PE (dominant), PP, PES	<0.5 mm, 0.5–1 mm, 1–5 mm (dominant), >5 mm	Filament and fiber (dominant), fragment	Transparent (dominant), black, white, green, red, blue	Muscle	7.16 ± 1.36	NR	FTIR-ATR	[22]
								Gut	2.16 ± 0.26	NR		
								Gill	2.66 ± 0.40	NR		
NR	Mackerel tuna	NR	Pangandaran East Coast, Indonesia	PP, PES	NR	Fragment (dominant), film, fiber, pellets	NR	Intestine	4.61 ± 2.61	NR	FTIR-ATR	[172]

*: Refers to all studied fish species, not specific to tuna. ** NR: Not Reported

## Data Availability

No data were used in this study.

## Abbreviations

ABS	Acrylonitrile butadiene styrene
ATR-FT-IR	Attenuated Total Reflectance FT-IR
EFSA	European Food Safety Authority
EPDM	Ethylene propylene diene monomer
EVA	Ethylene-vinyl acetate
FAO	Food and Agriculture Organization
FT-IR	Fourier-Transform Infrared Spectroscopy
GC-MS	Gas Chromatography-Mass Spectrometry
HDPE	High-density polyethylene
IR	Infrared
LDPE	Low-density polyethylene
LLDPE	Linear low-density polyethylene
MF	Melamine formaldehyde
Micro-FT-IR	Micro-Fourier Transform Infrared Spectroscopy
MPs	Microplastics
Mt	Million metric tons
PA	Polyamide
PAH	Polycyclic aromatic hydrocarbons
PAN	Polyacrylonitrile
PBDE	Polybrominated diphenyl ethers
PCBs	Polychlorinated biphenyls
PE	Polyethylene
PE-PP	Polyethylene-polypropylene copolymer
PET	Polyethylene terephthalate
PMMA	Polymethyl methacrylate
POPs	Persistent organic pollutants
PP	Polypropylene
PS	Polystyrene
PES	Polyester
PTFE	Polytetrafluoroethylene
PU	Polyurethane
PVA	Polyvinyl acetate
PVC	Polyvinyl chloride
ROS	Reactive oxygen species
SBR	Styrene-butadiene rubber
SEM	Scanning Electron Microscopy
UV	Ultraviolet
USFDA	United States Food and Drug Administration
UNEA	United Nations Environment Assembly
